# Thrombogenicity of stent graft materials as a potential contributing factor to limb graft occlusions

**DOI:** 10.1016/j.jvssci.2026.100436

**Published:** 2026-08-04

**Authors:** Job van Helvoirt, Hannah Jeffers, Michael Wolf, Hugo ten Cate, Carlijn Bouten, Simon Overeem, Erik Groot Jebbink, Michel Reijnen

**Affiliations:** a Multi-Modality Medical Imaging Group, Faculty of Science and Technology, University of Twente, Enschede, the Netherlands; b Cardiovascular Surgery, Medtronic Inc, Minneapolis, MN; c Global Technology and Innovation, Medtronic Inc, Minneapolis, MN; d Departments of Internal Medicine and Biochemistry, Maastricht University Medical Center, and Cardiovascular Research Institute (CARIM), Maastricht, the Netherlands; e Department of Biomedical Engineering, Soft Tissue Engineering and Mechanobiology Group, Eindhoven University of Technology, Eindhoven, the Netherlands; f Department of Vascular Surgery, Rijnstate Hospital, Arnhem, the Netherlands

**Keywords:** Thrombogenicity, Limb graft occlusions, In vitro blood analysis, Stent graft materials

## Abstract

**Objective:**

Limb graft occlusions (LGOs) remain a significant complication after endovascular aneurysm repair. Several clinical risk factors have been recognized, but these do not fully account for the consistently observed different LGO rates among major commercial stent graft systems. This paper investigates the thrombogenicity of stent graft materials as a potential factor for thrombus formation and therefore LGO.

**Methods:**

The thrombogenicity of the graft materials from three commercially available stent grafts (Zenith Alpha, Endurant, and Excluder) was evaluated in vitro using whole blood from four human donors for 1 hour in a dynamic setup. Samples were examined qualitatively with scanning electron microscopy, and the blood was further evaluated quantitatively via enzyme-linked immunosorbent assays for key indicators of thrombogenesis: normalized platelet count in the blood as measure of platelets in the clot, thrombin-antithrombin complex (TAT), β-thromboglobulin (βTG), and the soluble complement membrane attack complex (sC5b9).

**Results:**

Qualitative analysis revealed voluminous blot clot formation on Zenith Alpha stent grafts, some blood components on Endurant fabric, and minimal thrombotic factors on Excluder fabric. Material from the Zenith Alpha exhibited the lowest normalized platelet count in the blood (*P* < .0001) and significantly higher TAT (*P* < .001) and βTG values compared with Excluder (*P* = .002), indicating quantitatively the highest thrombogenicity among the evaluated stent graft materials. The Endurant fabric was significantly less thrombogenic compared with Zenith Alpha based on normalized platelet count (*P* < .001) and TAT values (*P* < .001). The Excluder graft material had the highest normalized platelet count (*P* < .0001) and lowest values of TAT (*P* < .0001) and βTG (*P* < .018), indicating that it is significantly less thrombogenic compared with the other two fabrics. No clear trend was found for sC5b9 between the stent graft materials.

**Conclusions:**

The materials used in three commercial stent graft systems have significantly different in vitro thrombogenic properties and thrombus formation on the fabrics.

**Clinical relevance:**

The found variations of material thrombogenicity between the Zenith Alpha, Endurant, and Excluder stent grafts appear to align with their reported clinical limb graft occlusion rates of these devices. By considering material thrombogenicity and thus thrombus formation, clinicians gain a deeper understanding of device-specific risks that may guide stent graft selection and postprocedural management. In general, these findings exemplify the potential relationship between implantable material properties and clinical complications, which remains insufficiently explored.

**Article Highlights**

• **Type of Research:** In vitro study
• **Key Findings:** In vitro testing with whole blood from four donors demonstrated significant device-specific differences in stent graft thrombogenicity. Qualitative (scanning electron microscopy) and quantitative analyses (platelet counts, thrombin-antithrombin complex, and β-thromboglobulin) identified Zenith Alpha as the most thrombogenic, Endurant as intermediate, and Excluder as the least thrombogenic.
• **Take Home Message:** Commercial endovascular aneurysm repair stent graft materials demonstrate significantly different thrombogenic properties, which may contribute to device-specific thrombus formation potentially influencing limb graft occlusion rates.

Endovascular aortic repair (EVAR) gained popularity because of its minimally invasive approach and good short-term and midterm outcomes.^1^ Despite its popularity, the durability of EVAR remains a topic of debate, largely due to the need for lifelong surveillance and secondary interventions associated with EVAR-related complications.^2^ A common complication after EVAR is limb graft occlusion (LGO), where thrombus formation inside the stent graft causes an occlusion in at least one of the stent limbs and/or the body of the endograft. LGO is a major cause of rehospitalization, reinterventions, and increased health care costs, and may result in limb-threatening conditions with an imminent risk of limb loss.^3^, ^4^, ^5^ Around 70% of LGOs occur within the first year after EVAR, generally affecting 0.7% to 12.4% of the patients.^4^, ^5^, ^6^ Different risk factors of LGO have been established, which are mostly dictated by the vascular anatomy. Identified factors are a small limb diameter <14 mm,^7^^,^^8^ iliac angulation >60°,^6^^,^^8^^,^^9^ calcification in both iliac arteries,^6^, ^7^, ^8^, ^9^ narrowed aortic bifurcation,^7^^,^^8^ and narrow distal landing zone in the external iliac artery.^4^^,^^10^ However, apart from anatomical factors, stent graft factors may also contribute to the risk on LGO. For example, graft material and design can fail to prevent limb kinking,^4^ which is related to tortuous anatomies,^8^^,^^10^ and excessive oversizing (>15%) may result in excessive infolding of fabric material.^6^^,^^8^ Research on the Anaconda stent graft (Terumo) showed high occlusion rates between 7.9% and 9.8%,^11^^,^^12^ which was linked to the stent's unique design, causing unfavorable hemodynamics due to the concertina effect.^13^ In another study, the use of Zenith Alpha was identified as an independent risk factor for developing an LGO with an incidence of 12.4%, whereas Endurant and Excluder showed LGO incidences of 3.4% and 0.7%, respectively, after a 5-year follow-up.^4^ Others published similar and consistent LGO rates between 9.0% and 12.4% for Zenith Alpha,^4^^,^^14^^,^^15^ between 2.4% and 4% for Endurant,^4^^,^^5^^,^^10^^,^^15^ and between 0.7% and 1.4% for Excluder.^4^^,^^16^ Cook Medical linked the high occlusion rates of their Zenith Alpha stent graft to limb misalignment in the main body, as stated in a 2020 urgent Field Safety Notice.^17^ Bogdanovic et al^4^ and Broda et al^14^ assessed the changes to the instructions for use highlighted in the Field Safety Notice and found that the factors mentioned were not over-represented in the occluded limbs of their studies, nor positively associated with the LGO incidence.

According to Virchow's triad, thrombosis results from disturbances in blood flow, pathological changes in blood composition, and interactions between blood and the vessel or artificial wall.^18^ Given that interactions between blood and artificial surfaces such as stent grafts affect thrombosis, differences in graft material may contribute to the device-specific LGO rates reported in the literature. The Zenith Alpha and Endurant fabrics are both composed of one layer, multifilament woven polyethylene terephthalate (PET), also known as Dacron, with a total thickness of around 100 μm and a fiber diameter of 12 μm. Although the fabrics may appear comparable, raw materials and manufacturing steps and processes might differ. The Excluder material is made from multiple layers of expanded polytetrafluorethylene (ePTFE) with a total thickness of around 100 μm. As material composition, surface characteristics, and manufacturing-related factors are known to influence blood-material interactions, these fabrics may exhibit different thrombogenic properties despite their apparent similarities.

The thrombogenicity of the fabrics is analyzed in accordance with ISO 10993-4 through quantification of platelets, thrombin-antithrombin complex (TAT), β-thromboglobulin (βTG), and the soluble membrane attack complex (sC5b9), and qualitative fabric analysis.^19^ Normalized platelet counts were used as a direct and objective measure of thrombus formation, providing a more reproducible assessment in this controlled in vitro setup compared with subjective surface-based thrombus quantification. TAT is a direct yet robust biochemical measure of the common coagulation pathway activation, with thrombin as the main catalyst for thrombus formation. The higher the TAT concentration, the more thrombogenic the material. βTG is a direct measure of platelet activation, contributing to mature clot formation. sC5b9 is the terminal complement complex, which is a link between thrombogenesis and inflammation response.

We hypothesized that stent grafts associated with higher clinical LGO rates exhibit greater thrombogenicity. Therefore, the objective of this study was to compare the in vitro thrombogenicity of the graft materials used in the Zenith Alpha, Endurant, and Excluder stent graft systems.

## Materials and methods

Thrombogenicity of three major stent graft fabrics, derived from the Zenith Alpha (Cook Medical), Endurant (Medtronic Inc), and Excluder (W.L. Gore & Assoc), respectively, was investigated by in vitro quantification of critical markers: normalized platelet counts, the platelet secretion marker βTG, the common coagulation pathway product TAT, and the sC5b9, representing the various cascades involved in thrombogenesis. In addition, clot formation on the fabrics was assessed qualitatively through macroscopic and microscopic inspection.

One stent per manufacturer was deployed in a sterile cleanroom, and the nitinol stent struts and sutures were removed in order to only investigate thrombogenicity of the fabric. Subsequently, the fabric was cut into 45 × 10-mm samples for the experiments. All experiments were performed in agreement with hemocompatibility test standards (ISO 10993-4:2017—Biological evaluation of medical devices—Part 4: Selection of tests for interactions with blood).^19^

Healthy blood donors were recruited using an institutional review board-approved protocol and consent form. Donors were screened using a standardized questionnaire to exclude individuals with known clotting disorders or the use of medications affecting coagulation or platelet function. Before enrollment, donors had to read and sign the consent form and meet eligibility criteria. Recruitment emails were distributed to current donors in accordance with the institutional review board protocol, and participation was granted for first responders. On the day of donation, all donors met the inclusion and exclusion criteria and signed and complied with the donation approval form.

Tubes were prepared containing a solution of 1 U/mL heparin diluted in PlasmaLyte (Baxter, Deerfield, IL), corresponding to the heparin concentration typically achieved during an EVAR procedure (5000 international units of heparin in 5 liters of blood). Thrombogenic borosilicate glass rods were added to the tubes as positive control (+control), whereas empty tubes with “no material” were used as negative control (−control).^19^ The fabric samples were placed randomly in the other tubes. Freshly drawn blood (1.5 mL) of consented, healthy, and anticoagulant-free donors (n = 4, three male and one female) was added to each tube of the material groups and control groups. Samples and controls were all tested in duplicate for each donor. To minimize potential bias associated with filling order, both tube filling and blood filling were randomized. Initial and final blood samples were collected to confirm for atraumatic and high-quality venipunctures. In clean, atraumatic blood draws, all markers should be similar in initial and final blood samples.

Once the tubes were filled with blood, they were placed on a rocker platform and incubated at 37 °C. The rocker motion roughly corresponded to a pulsatile flow of 60 pulses of 3.0 mL/min. This approach enables evaluation of material thrombogenicity, independent of different hemodynamic factors present in vivo, while preventing thrombosis formation due to stasis. After 1 hour of blood exposure, blood was withdrawn from each tube and further coagulation was prevented by quenching blood with CTAD anticoagulant (Greiner BioOne), a mixture of citrate, theophylline, adenosine, and dipyridamole. Nonadherent blood was removed from the samples by gentle rinsing with a controlled gravity-driven flow of PlasmaLyte buffer solution (Baxter). Macroscopic pictures of the samples were taken before fixing in Karnovsky's fixative (Polyscience Inc) for scanning electron microscopy (JOEL IT300LV ninja). Complete blood cell count data were acquired through measurement on a hematology analyzer (Beckman Coulter DxH 520), where platelet counts were recorded for thrombogenicity evaluation. To account for differences in baseline platelet counts between donors, samples were normalized to the average platelet count of the “no material” control, as per ASTM F2888 standard. Plasma supernatant was centrifuged, aliquoted, and subsequently analyzed in a technical duplicate for each sample with enzyme-linked immunosorbent assays to quantify TAT (Siemens Healthcare Diagnostics), βTG (Stago), and sC5b9 (Quidel Microvue) levels. The enzyme-linked immunosorbent assay data were generated in a blinded manner with respect to the material groups to minimize bias.

The normalized platelet counts, TAT, βTG, and sC5b9 data were compiled and analyzed in Minitab (State College) using analysis of variance to identify factors showing a statistically significant impact. A *P* value of <.05 was regarded as statistically significant. In case of significance, a Tukey's post hoc test was performed to test differences between groups.

## Results

Images of stent graft fabrics after 1 hour of blood exposure are shown in Fig 1. These images demonstrate a visible and marked difference among the three stent graft materials, with most thrombus formation on the Zenith Alpha material, followed by the Endurant material and then the Excluder material. Macroscopically, both PET materials (Zenith Alpha and Endurant) show increased blood adherence, whereas the ePTFE material of Excluder shows minimal blood coloration after rinsing.

**Fig 1:**
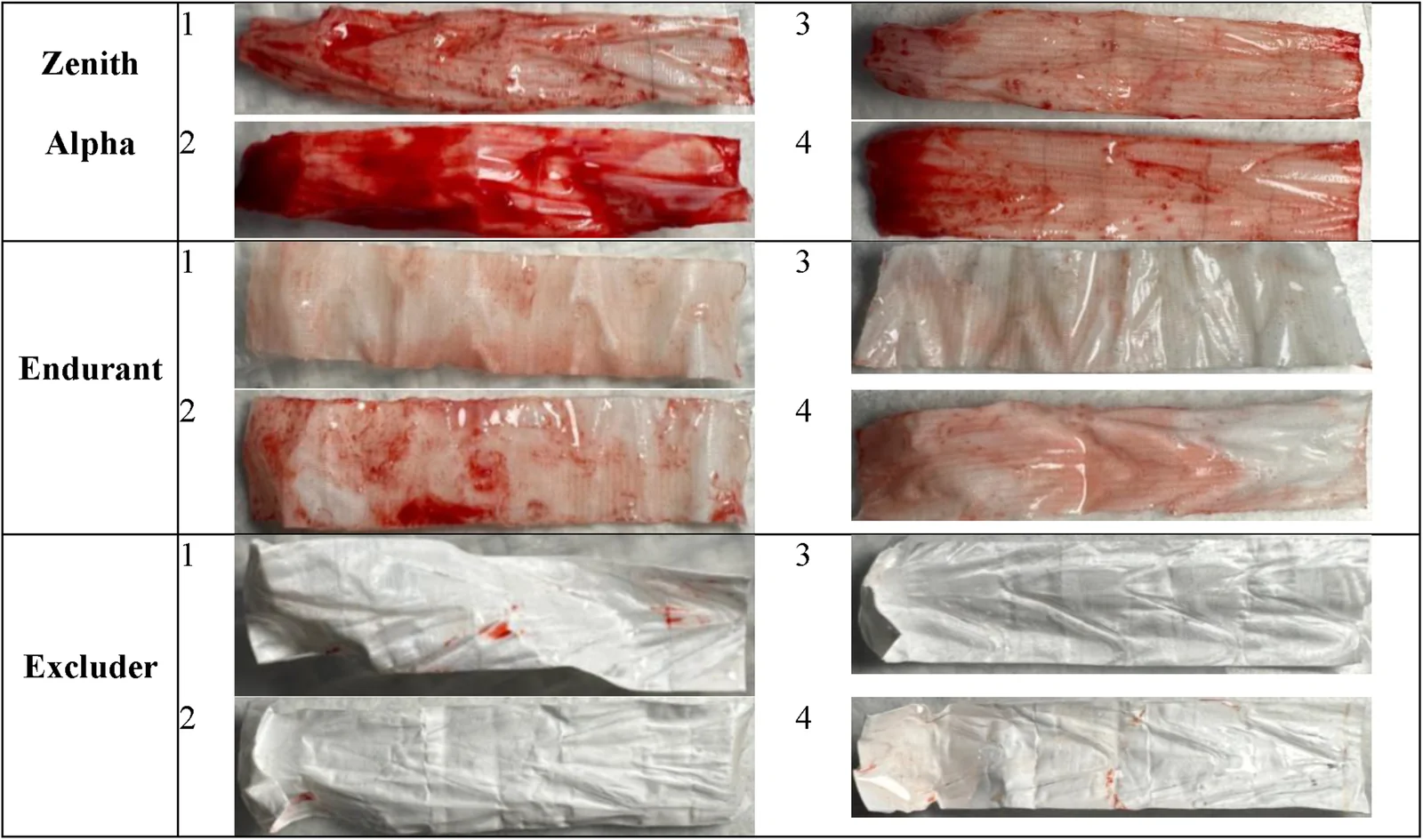
Representative pictures of different fabric samples (1 × 4.5 cm) after 1 hour of blood-contact and rinsing, of all donors (1-4).

Fig 2 shows the scanning electron microscopy images of the fabrics, with and without blood exposure on different magnifications, where distinct differences can be observed. The underlying structure of Zenith Alpha can still be recognized at 100× magnification; however, compared with the reference fabric, it is completely covered. At higher magnification (500×), the dense and voluminous fibrin network, with trapped red blood cells and platelets, can better be distinguished. Although the Endurant material (PET) and woven structure are similar to the Zenith Alphas fabric in the reference images, a visual difference in thrombus coverage between the exposed fabrics can be seen. The individual fibers of Endurant are still visible, with some red blood cells and platelets on the surface. Excluder is constructed using a different material, ePTFE, and it has a different structure as can be seen in the reference images. At higher magnification, some individual platelets and white blood cells can be distinguished between the typical nodal-and-fiber structure of ePTFE. However, the material is mainly free of blood components.

**Fig 2:**
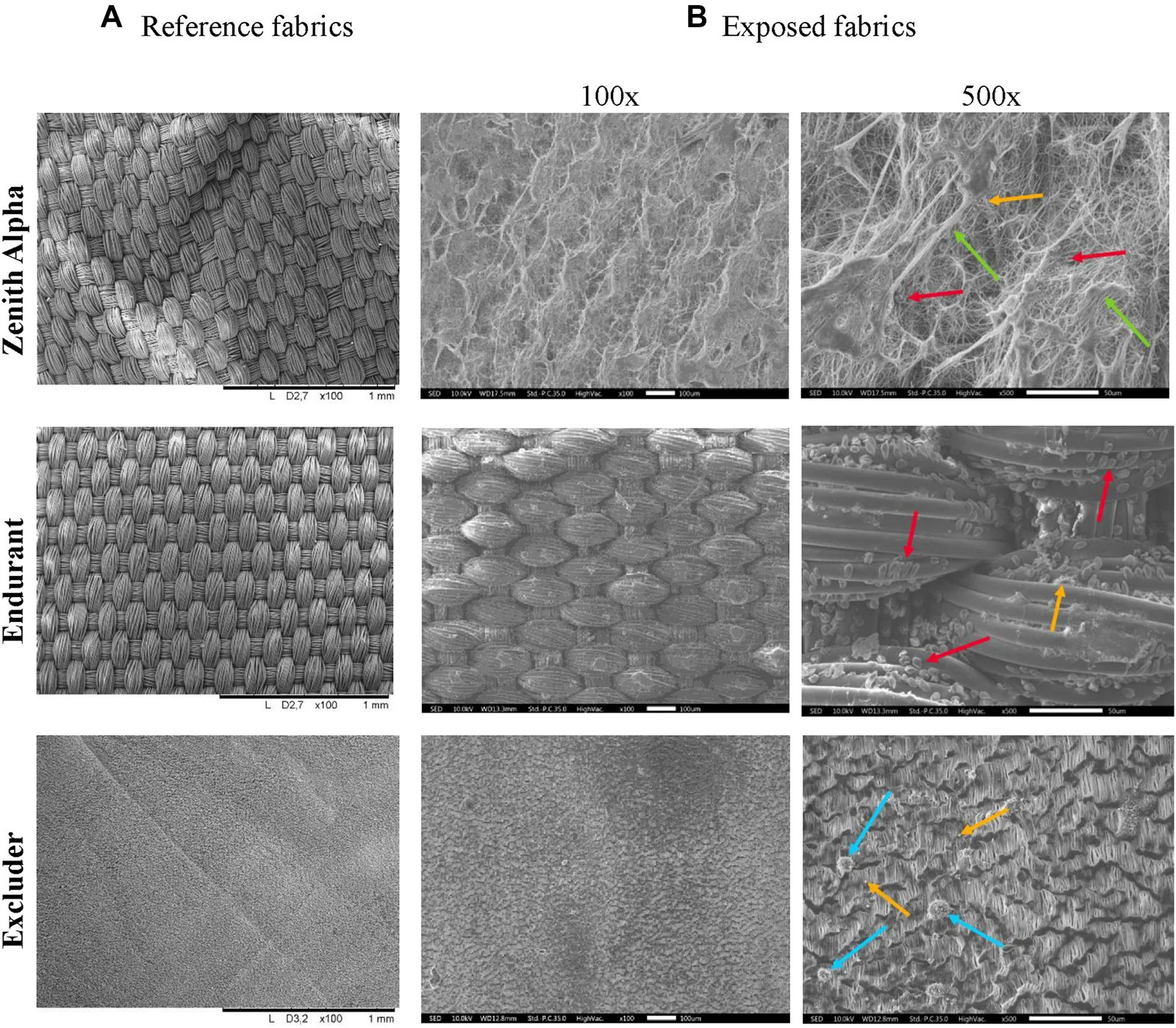
**A,** SEM images of the Zenith Alpha, Endurant, and Excluder reference fabrics. **B,** Representative SEM images of different fabric samples after 1 hour of blood contact and rinsing (donor A, 100× and 500× magnification, respectively). The *arrows* indicate (some) thrombotic factors such as fibrin (*green*), activated platelets (*orange*), red blood cells (*red*), and white blood cells (*blue*). *SEM*, Scanning electron microscopy.

The normalized platelet counts, shown in Fig 3, refer to the number of platelets remaining in the blood within the test tubes after exposure to the fabric samples or controls. Lower values indicate more platelet incorporation within the clot, thereby indicating a more thrombogenic material. Here, the positive control and Zenith Alpha have similar counts with significantly fewer platelets left in the blood compared with the other two materials and the negative control (*P* < .0001 for all). Excluder had significantly higher normalized platelet counts compared with Endurant (*P* = .018), and all materials had significantly lower normalized platelet counts compared with the negative control (*P* < .0001 for all).

**Fig 3:**
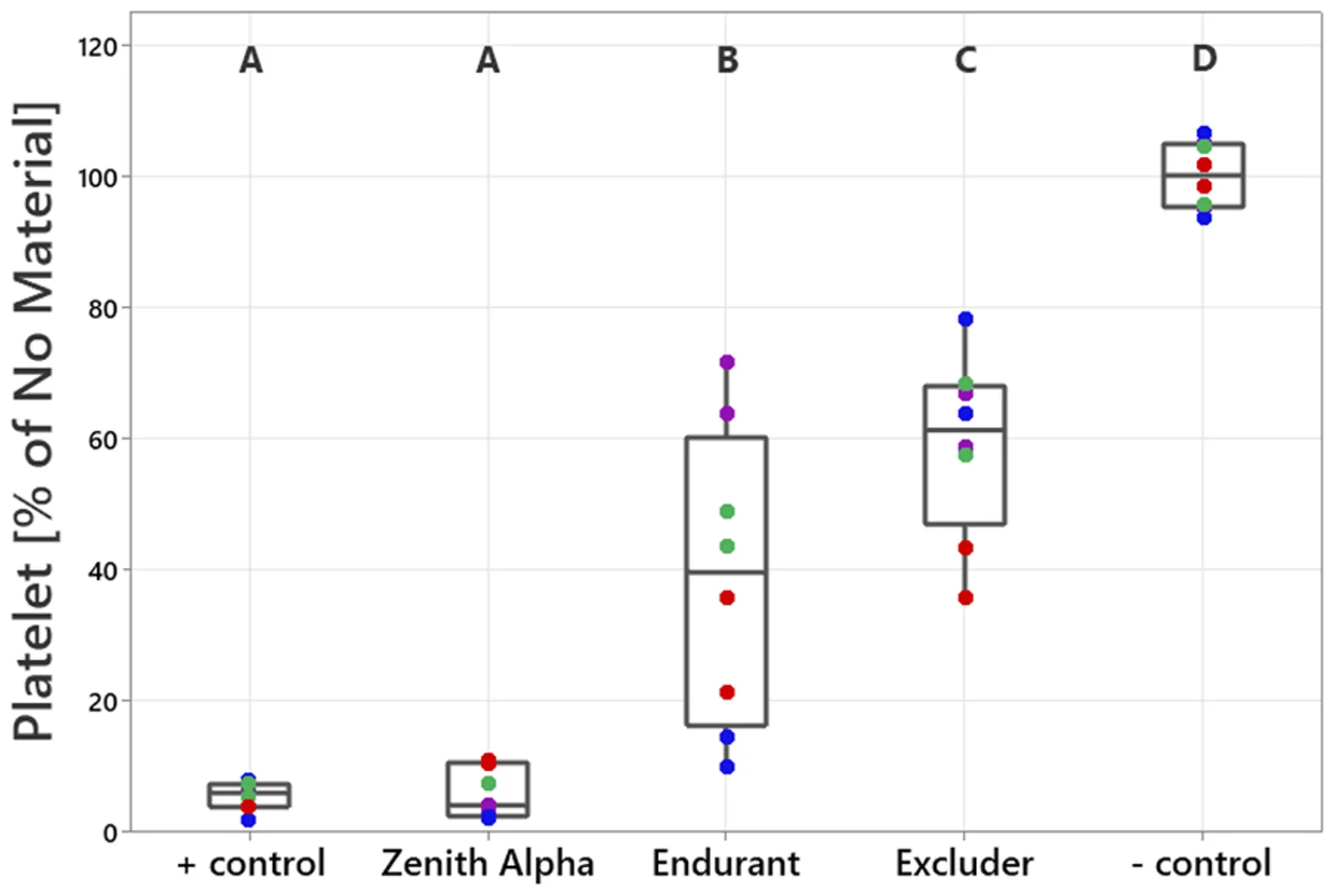
One-way analysis of normalized platelet counts as percentage of no material control by material. *Red* = donor 1, *blue* = donor 2, *green* = donor 3, and *purple* = donor 4. Groups with the same letter(s) are not significantly different (*P* ≥ .05), and groups with different letter(s) are significantly different (*P* < .05).

Results from the TAT analysis (Fig 4) indicate no significant difference between the Zenith Alpha material and the positive control (*P* = .28), whereas significantly lower TAT levels were found in the other groups (*P* < .001 for all). The TAT concentration in the blood with samples of the Excluder material was significantly lower than that from the Endurant graft material (*P* < .001). All materials were statistically more thrombogenic compared with the negative control (*P* < .001 for all).

**Fig 4:**
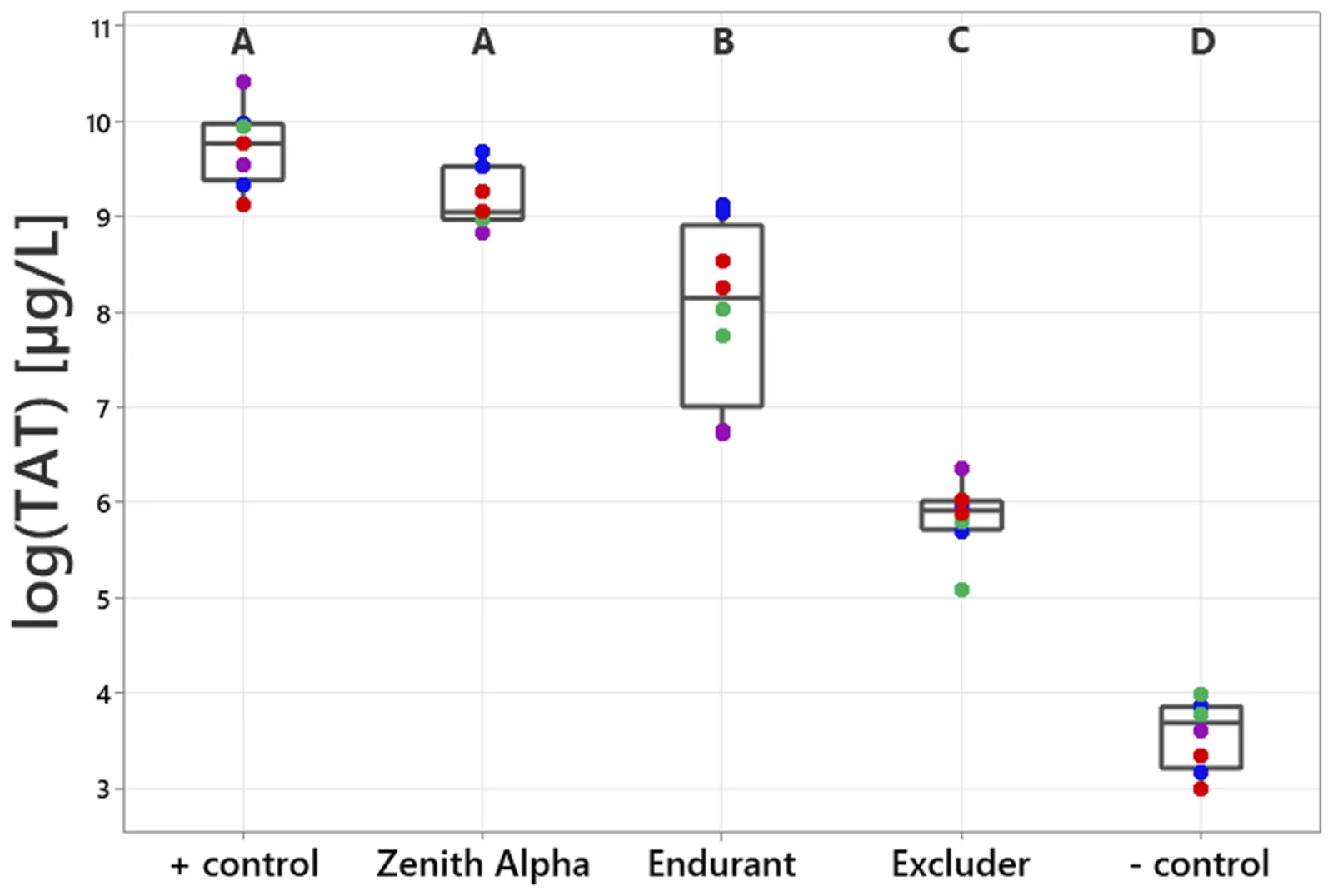
One-way analysis of log(TAT) in μg/L by material. *Red* = donor 1, *blue* = donor 2, *green* = donor 3, and *purple* = donor 4. Groups with the same letter(s) are not significantly different (*P* ≥ .05), and groups with different letter(s) are significantly different (*P* < .05). *TAT*, Thrombin-antithrombin complex.

Results from the βTG analysis (Fig 5) show a similar trend to the normalized platelet counts and TAT results, with decreasing βTG from Zenith Alpha to Endurant and then Excluder, although the level of significance varied from previous results. No statistically significant difference was found between the positive control and Zenith Alpha (*P* = .17), and Zenith Alpha and Endurant (*P* = .26). Excluder showed significantly less βTG concentration compared with both Zenith Alpha (*P* = .002) and Endurant (*P* = .031). Again, the negative control showed significantly less βTG compared with all materials (*P* < .0001 for all).

**Fig 5:**
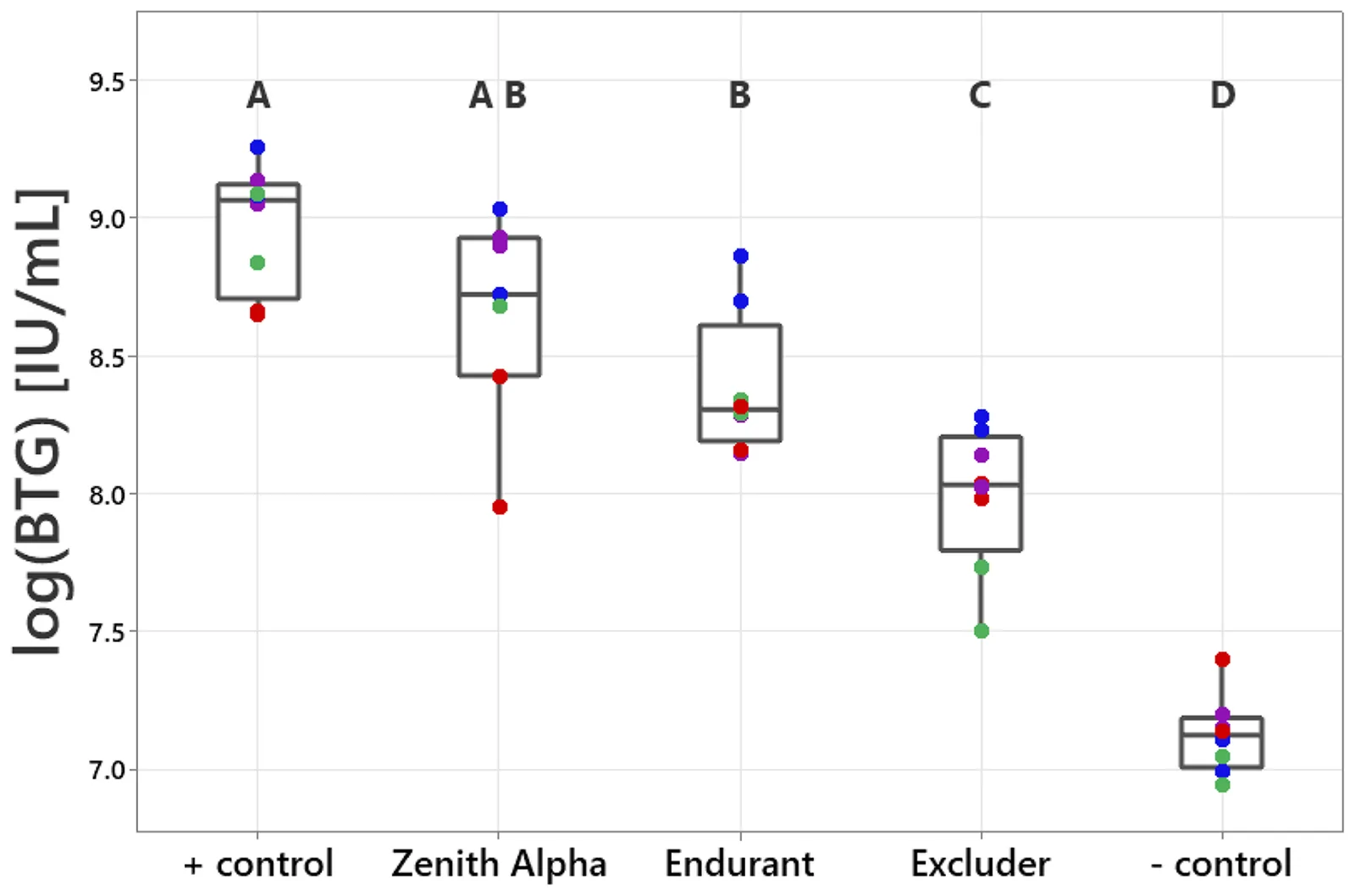
One-way analysis of log(βTG) in IU/mL by material. *Red* = donor 1, *blue* = donor 2, *green* = donor 3, and *purple* = donor 4. Groups with the same letter(s) are not significantly different (*P* ≥ .05), and groups with different letter(s) are significantly different (*P* < .05). *βTG*, β-Thromboglobulin.

The quantification of sC5b9, as shown in Fig 6, shows no trend. Only the Endurant material showed a significantly higher sC5b9 upregulation (*P* = .047) compared with the positive control.

**Fig 6:**
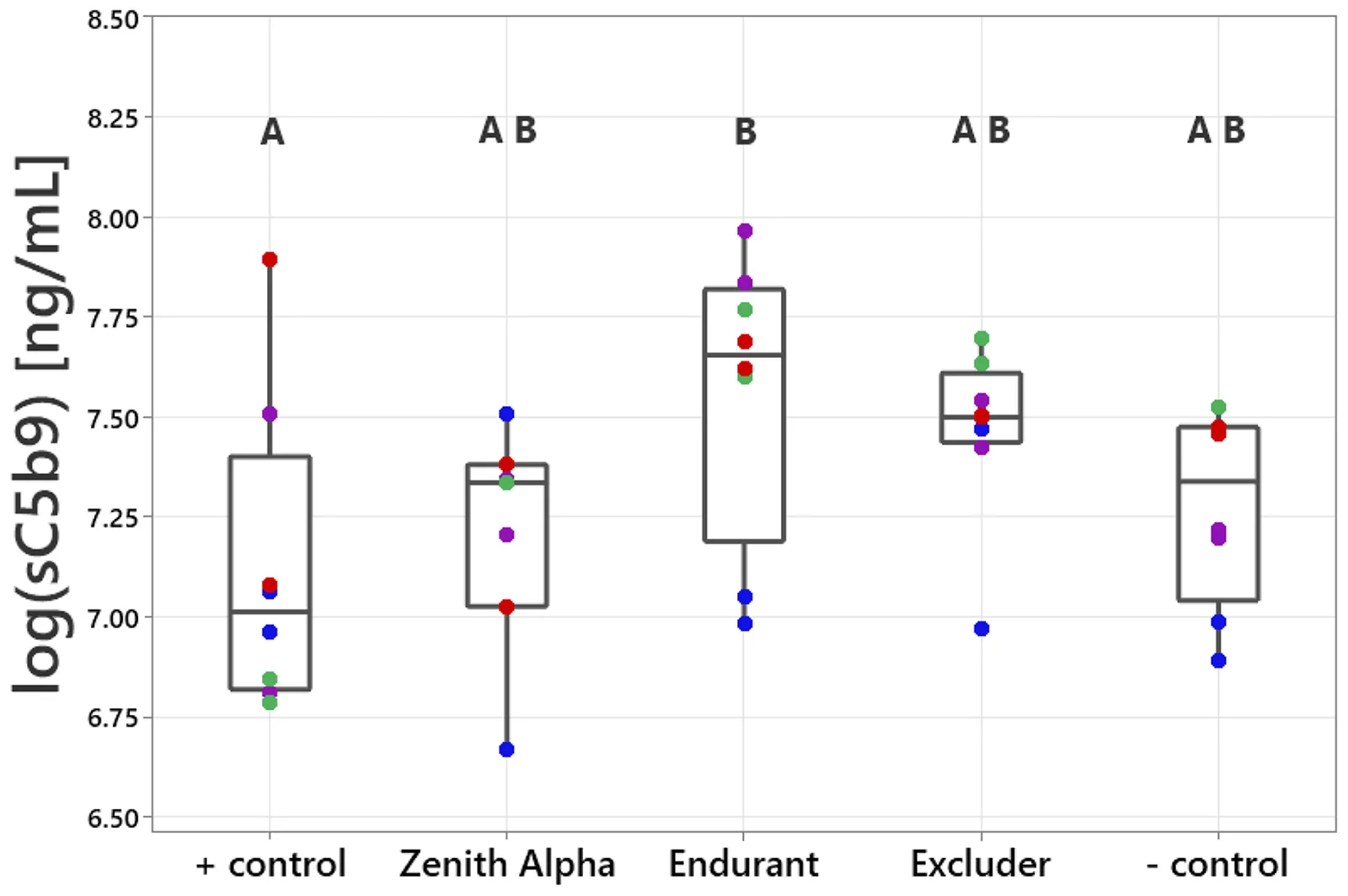
One-way analysis of log(sC5b9) in ng/mL by material. *Red* = donor 1, *blue* = donor 2, *green* = donor 3, and *purple* = donor 4. Groups with the same letter(s) are not significantly different (*P* ≥ .05), and groups with different letter(s) are significantly different (*P* < .05). *sC5b9*, Soluble membrane attack complex.

## Discussion

This study demonstrates marked differences in the in vitro thrombogenicity of widely used commercial stent graft materials. The results from this analysis, using platelet count, TAT, and βTG as markers, consistently demonstrated a thrombogenicity gradient among the three commercially available endografts, with Zenith Alpha showing the highest thrombogenicity, followed by Endurant, and Excluder being the least thrombogenic. This trend is comparable to published clinical LGO rates between these stent graft systems. sC5b-9 was included in accordance with ISO 10993-4 as a marker of complement activation and the broader innate inflammatory response linked to thrombogenesis. However, the limited differences observed suggest that complement-mediated effects may be less pronounced between the evaluated materials in this study. Zenith Alpha has been associated with the highest LGO rate^4^^,^^14^^,^^15^ of the three studied devices and was found in this research to be the most thrombogenic, comparable to the positive control. Literature has shown a lower LGO rate for Endurant,^4^^,^^5^^,^^10^^,^^15^ in line with the low to moderate thrombogenicity we observed in the current study, whereas the Excluder demonstrated the lowest thrombogenicity and is also associated with the lowest reported LGO rates.^4^^,^^16^ These findings suggest a possible association between the consistent LGO rates per device reported in the literature and the thrombogenicity of the corresponding fabric. It should be noted that there is limited clinical evidence regarding the independent contribution of graft material to LGO after adjustment for established anatomical and procedural risk factors. The present findings aim to provide a biological perspective that may help to further explain these observed differences and should be interpreted as complementary to, rather than replacing, established clinical predictors.

Similar thrombogenicity was expected for both PET-based fabrics (Zenith Alpha and Endurant), yet our results imply that, although bulk material might be identical (PET), minor variations at the blood-contacting surface can result in notable differences in thrombogenicity. Differences in surface characteristics might result from different raw PET polymer providers or different fabric manufacturing steps in the different companies. Residual spinning lubricants, textile processing aids, cleaning agents, sterilization-induced surface modifications, and other trace processing residues may alter surface chemistry and protein adsorption behavior. Such changes can influence thrombogenicity even if the bulk PET is identical. For example, hydrophobic fatty-acid esters and mineral-oil lubricants coming from spin finishes, drawing lubricants or weaving oils, make the surface more hydrophobic, which could promote and activate fibrinogen and platelets, causing a prothrombogenic effect.^20^ Another manufacturing-related effect could arise from the presence of polyethylene glycol, originating from spin finishes or emulsifiers, which may reduce fibrinogen absorption, platelet adhesion, and platelet activation through hydration-layer formation and steric repulsion, thereby contributing to an antithrombogenic effect.^21^ Significantly lower LGO rates have been reported for the ZSLE limbs (Zenith Flex platform) compared with the newer Zenith Alpha platform.^22^ Interestingly, this observation may be consistent with the concept that manufacturing-related differences can influence biological responses, even when the base material remains PET. Although both devices use PET-based graft fabrics and come from the same manufacturer, the fabrics differ substantially in architecture, thickness, fiber density, and surface morphology, inherently requiring different manufacturing and processing approaches. Hypothetically, such differences may contribute to increased thrombogenicity of the Zenith Alpha fabric and thereby to the higher reported LGO rates compared with its predecessor platform, Zenith Flex. In light of the current study, it could be hypothesized that the Zenith Flex fabric is less thrombogenic than the Zenith Alpha fabric; however, this hypothesis remains untested and warrants further investigation. All this indicates that these more elaborate blood-material analyses may be relevant in future stent graft development and manufacturing process changes.

Both macroscopic and microscopic images clearly represented the same trend as seen in the normalized platelet counts, TAT, and βTG results. Although Excluder showed the lowest thrombogenicity among the tested devices, all thrombogenic markers remained significantly higher than those in the negative control; nevertheless, little to no thrombus was observed after rinsing. Because the biomarkers reflect activation of elements before actual thrombus formation, it is possible that the thrombogenic drivers remained below a certain threshold in the least thrombogenic materials. In the absence of artificial surfaces, such a situation has been referred to as “prethrombotic state.”^23^ Surface properties influencing platelet and fibrin deposition might explain the low amount of thrombus seen on the Excluder surface; this could imply that the prothrombotic factors that were quantified did not immobilize on the surface but are still activated and in the blood. Whether such a prethrombotic state has clinically relevant implications remains insufficiently explored, and the biological and clinical significance of this potential phenomenon in ePTFE materials therefore remains uncertain. Both the reported LGO incidences and the present in vitro findings indicate meaningful differences in thrombotic risk among commercially available stent grafts. Although the results of this study should be interpreted with caution, the observed differences in LGO rates between devices raise the clinically relevant question of whether postoperative antithrombotic therapy should be tailored to specific graft types. Currently, however, there is still insufficient evidence to change clinical practice. Current European Society for Vascular Surgery (ESVS) guidelines recommend single antiplatelet therapy after EVAR; prospective studies are needed to determine whether device-specific antithrombotic strategies can reduce LGO without increasing bleeding risk. Generally, these results indicate that there is a measurable difference in in vitro biological response to various materials, which may have additional clinical implications warranting further discussion and investigation. A study by Kuijpers et al^24^ showed that ePTFE grafts have a significantly higher prevalence of type 2 endoleaks compared with PET grafts. Another retrospective analysis from Nana et al^25^ also linked reduced type 2 endoleaks, as well as increased sac regression to the use of PET rather than ePTFE stent grafts. Although these publications do not elaborate on how these materials influence the clinical outcomes, they highlight the importance and potential of using different materials. The concept of incorporating thrombogenic material on the outer surface of a stent graft to promote thrombus formation and potentially reduce type 2 endoleaks has also been explored successfully in vitro by Chaput et al.^26^ In current research, both PET materials (Zenith Alpha and Endurant) were more thrombogenic compared with the ePTFE material. Hypothetically, these PET materials could induce more thrombus formation in the aneurysmal sac, lumbar arteries, and/or inferior mesenteric artery, naturally avoiding backflow, decreasing type 2 endoleak prevalence. Conversely, less thrombogenic materials are unlikely to actively promote type 2 endoleaks but may be less effective in preventing them. Reduced type 2 endoleaks could potentially lead to better sac regression rates and improved overall outcomes of EVAR.^27^ Although thrombogenic materials may adversely impact the luminal side of stent grafts by promoting thrombus formation and LGO, further research could shed light on the effect of material-induced thrombus formation on the outside of the stent graft and its relation to type 2 endoleaks and sac regression.

Also, other clinical observations could be linked to thrombogenicity. Postimplant syndrome (PIS) is represented by malaise, fever, and increased inflammation markers, and this phenomenon is more often observed in patients treated with a PET stent graft compared with ePTFE.^28^ Fundamentally, thrombogenesis and inflammation are closely intertwined.^29^ Material thrombogenicity might upregulate inflammatory markers, also known as thromboinflammation,^30^ which could be clinically represented as PIS.

Although an association between PIS, lower type 2 endoleak rate, and sac regression has been proposed before, direct causation is unconfirmed.^31^ Material selection is known to affect biological responses in fields such as orthopedics, wound healing, and cardiovascular diseases.^32^, ^33^, ^34^ Further research should examine how stent graft materials impact EVAR outcomes, potentially enabling tailored material choices to address clinical challenges, such as combining different materials within a single graft to elicit targeted biological and clinical effects.

Several limitations should be acknowledged. The study featured a relatively small sample size (n = 4 donors tested in duplicate for every material) and three graft materials. Although typical for similar experimental designs, it may restrict the generalizability of the outcomes. The donor cohort comprised healthy individuals (three males and one female), potentially lacking blood characteristics representative of patients with aneurysm due to variations in age, disease state, or medication use. No antiplatelet or anticoagulant therapy was administered to the donors in order to assess the intrinsic thrombogenicity of the stent graft materials, independent of pharmacological influences. Although this approach is consistent with standardized in vitro hemocompatibility testing, future studies should investigate the effect of antithrombotic regimens on material thrombogenicity given their clinical relevance for EVAR patients. Technically, the experimental protocol ensured consistent blood movement through rocking to prevent clotting because of stasis. Although this is suitable for investigating blood-material interactions such as thrombogenicity, it does not completely recapitulate physiological flow conditions observed in vivo. It should be noted that thrombogenicity itself is an intrinsic material characteristic rather than a phenomenon influenced by in vivo hemodynamics that has its own influence on thrombus formation. The experiments were conducted within a 1-hour time frame in vitro, and although this time window allowed for the observation of differences between blood and material responses, it will not capture all aspects of in vivo thrombus formation, maturation, and lysis. Moreover, although various markers were evaluated to investigate thrombogenesis, fibrin formation was only qualitatively analyzed through visualization. Given the interconnected nature of thrombogenesis pathways, consistent trends observed across quantifications may indicate underlying biological relationships rather than entirely separate processes. In addition, this study does not address long-term biological responses on the graft surface. Although this in vitro study provides a controlled assessment of material-blood interactions, translation of these findings to clinical outcomes remains challenging, as no established in vivo model fully captures the complex interplay between graft material, hemodynamics, and patient-specific factors. Lastly, a more detailed investigation of surface characteristics and manufacturing processes may help elucidate the underlying causes of the observed differences in thrombogenicity between the Zenith Alpha and Endurant devices. However, such analyses may be challenging, as key aspects of device design and production are often proprietary and not publicly accessible.

## Conclusions

Our findings reveal significant differences in the in vitro thrombogenicity of three commercial stent graft materials—namely Zenith Alpha, Endurant, and Excluder. These variations appear to align with reported clinical LGO rates of these devices, suggesting that material thrombogenicity might play a contributory role to this clinical complication. Future research should focus on elucidating the mechanisms underlying stent graft thrombogenicity, further characterizing the role of hemodynamics in LGO, and prospectively evaluating device-specific outcomes in a patient-matched population to better understand the contribution of graft-related factors to LGO.

## Author Contributions

Conception and design: JH, HJ, MW

Analysis and interpretation: JH, HJ, MW, HC, CB, SO, EJ, MR

Data collection: JH, HJ

Writing the article: JH, HJ

Critical revision of the article: MW, HC, CB, SO, EJ, MR

Final approval of the article: JH, HJ, MW, HC, CB, SO, EJ, MR

Statistical analysis: Not applicable

Obtained funding: Not applicable

Overall responsibility: JH

EJ and MR shared senior authorship

## Funding

The research was conducted using resources of 10.13039/100004374Medtronic.

## Disclosures

J.V.H., H.J., M.W., and S.O. are employed at Medtronic. M.R. received consulting fees and research grants from W. L. Gore & Associates, Medtronic, and Terumo Aortic. E.G.J. received research grants from W. L. Gore & Associates, Medtronic, and Terumo Aortic. The remaining authors report no conflicts.
